# Age-associated differences in cervical sagittal alignment parameters among asymptomatic adults: a cross-sectional reference study

**DOI:** 10.3389/fnins.2026.1856814

**Published:** 2026-08-05

**Authors:** Yu Zhang, Chong Zhao, Chaoyang Huang, Jie Huang, Hongzhen Li, Xinjie Li, Junjie Ruan, Haiying Liu, Shuai Xu

**Affiliations:** 1 Department of Spinal Surgery, The People’s Hospital of Yudu County, Ganzhou, Jiangxi, China; 2 Department of Spinal Surgery, Peking University People’s Hospital, Peking University, Beijing, China; 3 School of Clinical Medicine, Peking University Health Science Center, Peking University, Beijing, China; 4 Department of Orthopaedics, Beijing Tsinghua Changgung Hospital, Beijing, China

**Keywords:** cervical sagittal vertical axis, cervical spine, neck tilt, reference values, sagittal alignment, TIA-CL

## Abstract

**Purpose:**

While age-adjusted alignment goals are established for the lumbar spine, analogous clinically actionable tools are lacking for cervical sagittal assessment. This study aimed to derive and internally assess simple, age- and sex-specific formulas for key cervical parameters—thoracic inlet angle minus cervical lordosis (TIA-CL), neck tilt (NT), and cervical sagittal vertical axis (cSVA)—in asymptomatic adults.

**Methods:**

This retrospective cross-sectional study analyzed 632 rigorously screened asymptomatic adults (268 males, 364 females; mean age 54.8 ± 16.6 years) who underwent neutral lateral cervical radiography. Radiographic parameters (TIA, C2–C7 lordosis, NT, cSVA) were measured using a validated protocol. All angular parameters (TIA, CL, TIA-CL, and NT) are reported in degrees (°), whereas cSVA is reported in millimeters (mm). Sex-stratified linear regression models quantified age-related associations for TIA-CL, NT, and cSVA, with internal resampling via 1,000-iteration bootstrap resampling (CI width < 0.15 indicating good stability).

**Results:**

All three parameters showed significant positive associations with age (all *P* < 0.05). The derived simplified formulas were: TIA-CL ≈ 56 + 0.07 × Age (R^2^ = 0.010), NT ≈ 40 + 0.16 × Age (R^2^ = 0.111), and cSVA ≈ 15 + 0.10 × Age (R^2^ = 0.045). Age-associated differences were more pronounced for NT before age 50 (*P* = 0.005) and for cSVA after age 50 (*P* = 0.028). Males had significantly higher baseline NT and cSVA than females (*P* = 0.009 and *P* < 0.001). Bootstrap resampling demonstrated internal consistency of the model estimates (all 95% CI widths < 0.15).

**Conclusion:**

We provide age- and sex-specific reference formulas for NT and cSVA, and an age-specific formula for TIA-CL. These intuitive equations offer a practical reference for comparing an individual’s alignment against age-associated population estimates.

## 1 Introduction

Cervical sagittal alignment is a critical determinant in the preoperative evaluation and postoperative outcome prediction for patients with cervical disorders, as it directly influences spinal biomechanics, load distribution, and neurological function (Martini et al., 2021; Patwardhan et al., 2018). Clinically, parameters such as cervical sagittal vertical axis (cSVA) and C2–C7 lordosis (CL) are widely measured, with static thresholds (e.g., cSVA > 40 mm) often employed to define pathological malalignment (Caffard et al., 2024). However, a growing body of evidence, including from our own team, underscores that these “normative” values are not static but exhibit significant age-associated differences (Ani et al., 2025; Lee et al., 2022, 2023). This reliance on fixed thresholds overlooks a fundamental biological variable, potentially leading to the misclassification of physiological aging as pathology or, conversely, the underdiagnosis of significant imbalance in younger individuals (Evaniew et al., 2022; Liu et al., 2023).

The paradigm for age-adjusted assessment is well-established in the lumbo-pelvic spine. Inspired by the foundational work of Schwab et al., which identified pelvic incidence minus lumbar lordosis (PI-LL), pelvic tilt (PT), and sagittal vertical axis (SVA) as key alignment targets (Hey et al., 2023; Passias et al., 2021), Lafage et al. (2017) subsequently derived practical, age-specific formulas (e.g., PI-LL = [Age−55]/2 + 3) that have been widely adopted to guide surgical planning in adult spinal deformity. This successful translation of complex biomechanical principles into simple clinical references highlights a critical gap in cervical spine care: while the age-associated differences is acknowledged, a simple, formula-based reference—analogous to that used in the lumbar spine—is conspicuously absent.

This gap is particularly pertinent given the established biomechanical analogy between the cervical-thoracic and lumbo-pelvic junctions. Recent studies have validated the thoracic inlet angle (TIA) as a relatively invariant anatomical parameter analogous to PI, with neck tilt (NT) and T1 slope (T1S) paralleling PT and sacral slope (SS), respectively (Lee et al., 2012; Takakura et al., 2024). The fundamental geometric relationship is TIA = NT + T1S, which mirrors the pelvic relationship PI = PT + SS (Baker, 2023). This structural analogy provides a coherent framework for assessing regional balance.

Furthermore, just as PI-LL serves as a mismatch parameter representing the discrepancy between the fixed anatomical angle (PI) and the modifiable lumbar curvature (LL) in the lumbopelvic complex, the corresponding cervical mismatch parameter can be defined as TIA-CL (or alternatively T1S-CL). Both parameters reflect the balance between an inlet/anatomical angle and regional lordosis, with larger values indicating greater compensatory demand or potential imbalance. In the present study, TIA-CL was selected as the primary mismatch parameter because it incorporates both NT and T1S, thereby providing a more comprehensive measure of cervicothoracic compensatory reserve than T1S-CL alone.

Preliminary work has confirmed cross-sectional associations between these cervical parameters and age (Been et al., 2017) yet these studies have primarily described trends without delivering the quantitative, simplified models required for direct clinical application. To bridge this gap between descriptive research and clinical utility, the present study had two primary objectives: (1) to derive and validate simple, age- and sex-specific linear formulas for the key cervical sagittal balance parameters (TIA-CL, NT, and cSVA) in a large cohort of asymptomatic adults, thereby establishing population-level reference estimates; and (2) to perform comprehensive subgroup analyses to explore sex- and age-stratified patterns. Ultimately, we aim to provide a set of internally validated equations that offer age-associated reference values for cervical sagittal alignment, which may serve as a foundation for future clinical studies.

## 2 Materials and methods

### 2.1 Study population and data source

This retrospective cross-sectional study was conducted using data from the health screening cohort of Peking University People’s Hospital. Between January 2016 and January 2020, individuals undergoing comprehensive annual health examinations, which included a standing lateral cervical radiograph as part of a systematic spinal evaluation protocol, were considered for inclusion. The primary purpose of this radiographic screening was to identify undiagnosed spinal conditions in an asymptomatic population. This study was approved by the institutional review boards of our institution (approval number: 2024PHB100-001) and conducted in accordance with the Declaration of Helsinki. All participants provided general consent for their anonymized data to be used for medical research.

Eligible participants were aged 20–92 years with no self-reported neck or back pain (Neck Disability Index, NDI score < 3), no clinical history suggestive of cervical spine pathology (e.g., radiculopathy, myelopathy), and had intact neutral lateral cervical radiographs. Subjects were excluded if they met any of the following criteria: (1) congenital or acquired cervical fusion; (2) spinal deformity (e.g., scoliosis, kyphoscoliosis); (3) unclear visualization of the T1 superior endplate or C7 inferior endplate on radiographs; (4) history of spinal surgery, trauma, tumor, or infection; (5) systemic spinal diseases (e.g., ankylosing spondylitis, rheumatoid arthritis); or (6) incomplete demographic or radiographic data. A total of 632 participants were included in the final analysis after applying the inclusion and exclusion criteria.

### 2.2 Data collection and radiographic measurements

Demographic data collected included age and sex. All lateral cervical radiographs were obtained in a standardized neutral weight-bearing position: participants stood upright with feet shoulder-width apart, arms naturally hanging at the sides, eyes looking straight ahead, and cervical spine in a relaxed neutral posture (Baker, 2023). Radiographic parameters were measured twice independently by two trained authors using a picture archiving and communication system (PACS, Neosoft, CN), and the average value of the two measurements was used for analyses. Reliability data (intraclass correlation coefficient, ICC range 0.81–0.92) are from the original report Zhang et al. (2024) and were not recalculated for the present analysis, which is a secondary analysis of the same cohort focusing on deriving age-associated reference formulas that have not been previously reported.

The measured parameters included: CL, TIA, NT, and cSVA. While TIA and NT are not as routinely measured as cSVA or CL, they were included based on their validated role in the cervical-thoracic balance analogy and their potential to provide a more comprehensive assessment of compensatory mechanisms (Takakura et al., 2024). The key cervical cervicothoracic balance parameters, analogous to the lumbo-pelvic parameters PI-LL, PT, and SVA, were TIA-CL, NT, and cSVA.

**FIGURE 1:**
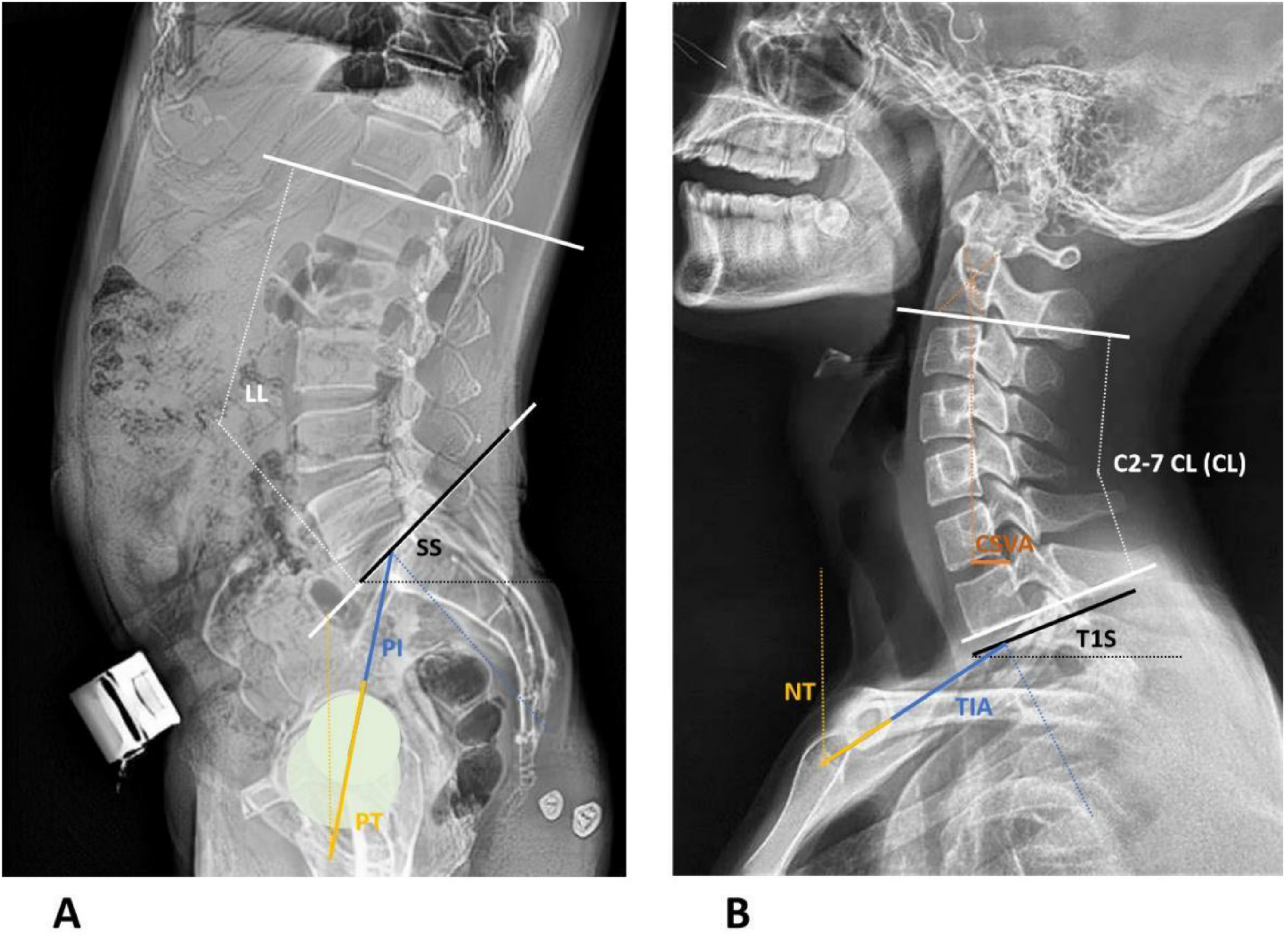
Radiographic measurements of sagittal spinal parameters. **(A)** Lumbar spine and pelvis: LL (lumbar lordosis), SS (sacral slope), PI (pelvic incidence), PT (pelvic tilt). **(B)** Cervical and upper thoracic spine: C2-7 CL (cervical lordosis, defined as the angle between the superior endplate of C2 and the inferior endplate of C7), cSVA (cervical sagittal vertical axis, defined as the horizontal distance between the posterior superior corner of C2 and a vertical plumb line dropped from the posterior superior corner of C7), T1S (T1 slope, defined as the angle between the superior endplate of the T1 vertebra and a horizontal reference line), TIA (thoracic inlet angle, defined as the angle between the line parallel to the T1 superior endplate and the line connecting the T1 superior endplate to the center of the sternoclavicular joint), NT (neck tilt, defined as the angle between the vertical line and the line connecting the T1 superior endplate to the center of the sternoclavicular joint).

All angular parameters were measured in degrees (°), and cSVA was measured in millimeters (mm). The following definitions were applied (Figure 1):

CL: The Cobb angle between the superior endplate of C2 and the inferior endplate of C7. Lordosis (anterior convexity) was recorded as a positive value.
TIA: The angle between a line parallel to the superior endplate of T1 and a line connecting the midpoint of the T1 superior endplate to the center of the sternoclavicular joint.
NT: The angle between the vertical reference line and a line connecting the midpoint of the T1 superior endplate to the center of the sternoclavicular joint.
T1S: The angle between the superior endplate of T1 and the horizontal reference line.
TIA-CL: Calculated as TIA minus CL. This parameter represents the mismatch between the thoracic inlet angle and cervical lordosis.
cSVA: The horizontal distance between the posterosuperior corner of the C2 vertebral body and a vertical plumb line dropped from the posterosuperior corner of C7. A positive value indicates anterior translation of C2 relative to C7.

In addition, males and females were analyzed separately to evaluate sex-specific differences. Additionally, the 50-year cutoff was selected based on prior literature suggesting midlife (approximately age 50) as a period of accelerated spinal degenerative differences (Dandumahanti and Subramaniyam, 2024; Wang et al., 2023). This threshold was prespecified before subgroup analyses.

### 2.3 Statistical analysis

Statistical analyses were performed using SPSS version 24.0 (SPSS Inc., Chicago, United States) and R software version 4.3.2 (R Foundation for Statistical Computing, Vienna, Austria). Continuous data are presented as mean ± standard deviation (SD) or median (interquartile range) as appropriate. Categorical data are presented as counts and percentages.

Demographic characteristics and radiographic parameters were summarized for the total population and subgroups. Differences between two subgroups were examined using independent-samples *t*-tests (continuous variables). Pearson correlation coefficients were calculated to assess the linear relationships between age and each balance parameter (TIA-CL, NT, and cSVA). Linear regression models were constructed to quantify the associations between age and the three key parameters for clinical practicality. Model performance was evaluated using the coefficient of determination (R^2^) and F-statistic. Although higher-order (quadratic and cubic) models were tested for comparison, linear models were ultimately selected and reported for the primary analyses to maximize clinical interpretability and utility.

Bootstrap resampling (1,000 iterations) was performed to assess the internal stability of the regression model estimates. The median R^2^ and its 95 % confidence intervals (CIs) from bootstrap samples were reported, with a CI width < 0.15 indicating acceptable internal consistency (Robbins and Burgette, 2025). Comprehensive model diagnosis was conducted to verify regression assumptions, including normality of residuals (Shapiro-Wilk test), homoscedasticity (Breusch-Pagan test), and absence of multicollinearity (variance inflation factor, VIF, <2 considered acceptable). Outliers were identified using the 3σ rule (residuals > 3 SD from the mean), with an outlier rate < 5% considered acceptable for large samples. Interaction terms (Age × Sex, Age × Age Group) were included in regression models to test for differences in age-related slopes between subgroups. Akaike information criterion (AIC) and Bayesian information criterion (BIC) were used to compare model fit, with smaller values indicating better balance of fit and parsimony. Linear regression models were prespecified as the primary analytic approach to maximize clinical interpretability and provide easily memorable reference formulas, consistent with the study’s aim. Higher-order (quadratic and cubic) models were explored for sensitivity analysis and model comparison, but were not intended as the primary presentation.

## 3 Results

A total of 1,572 individuals who underwent cervical spine lateral radiography between January 2016 and January 2020 were initially screened for eligibility. After strict application of predefined exclusion criteria, 632 asymptomatic subjects (268 males and 364 females) were included. Demographic characteristics and cervical sagittal parameters of the study population are detailed in Table 1. The overall mean age of participants was 54.8 ± 16.6 years (range: 20–92 years). ICC values ranged from 0.81 to 0.92, indicating good to excellent reliability of the radiographic measurement protocol, consistent with our previous report (Zhang et al., 2024).

**TABLE 1:** Demographic and clinical characteristics (by sex).

Variable	Total population *(n* = 632)	Male (*n* = 268)	Female (*n* = 364)	*P*-value
Age, years	54.8 ± 16.6 (20–92)	54.0 ± 17.1 (20–92)	55.4 ± 16.2 (20–91)	0.328
TIA, °	78.2 ± 14.2 (40.5–113.3)	76.1 ± 14.0 (40.5–113.3)	79.8 ± 14.3 (44.1–113.3)	0.036*
C2–C7 CL, °	17.5 ± 14.8 (−31.0 to 53.1)	15.8 ± 15.1 (−31.0 to 48.6)	19.6 ± 13.5 (−23.1 to 53.1)	0.124
TIA-CL, °	60.4 ± 12.0 (14.2–99.2)	61.2 ± 12.7 (15.3–99.2)	59.7 ± 11.4 (14.2–98.7)	0.087
NT, °	48.7 ± 7.74 (26.1–76.3)	49.7 ± 7.51 (28.5–76.3)	47.9 ± 7.85 (26.1–74.9)	0.009**
T1S, °	31.7 ± 9.6 (0–64.8)	30.8 ± 9.6 (0–64.8)	32.7 ± 9.6 (6.3–64.9)	0.865
cSVA, mm	20.2 ± 9.68 (−6.4 to 52.3)	22.1 ± 11.04 (−6.4 to 52.3)	18.9 ± 8.56 (−5.8 to 49.8)	<0.001***

Data are presented as mean ± standard deviation (range) for continuous variables. TIA, thoracic inlet angle; C2–C7 CL, C2–C7 cervical lordosis; TIA-CL, TIA minus C2–C7 (CL); T1S, T1 slope; cSVA, cervical sagittal vertical axis

*P < 0.05,

**P < 0.01,

***P < 0.001.

The mean values of core parameters were CL of 17.5 ± 14.8°, TIA-CL of 60.35 ± 12.01°, NT of 48.68 ± 7.74°, T1S of 31.7 ± 9.6°, and cSVA of 20.23 ± 9.68 mm. Males had a higher TIA, NT and cSVA than females (*P* = 0.036, *P* = 0.009, and *P* < 0.001), while no statistically significant sex differences were found in CL (*P* = 0.124) or TIA-CL (*P* = 0.087) (Table 1).

**FIGURE 2:**
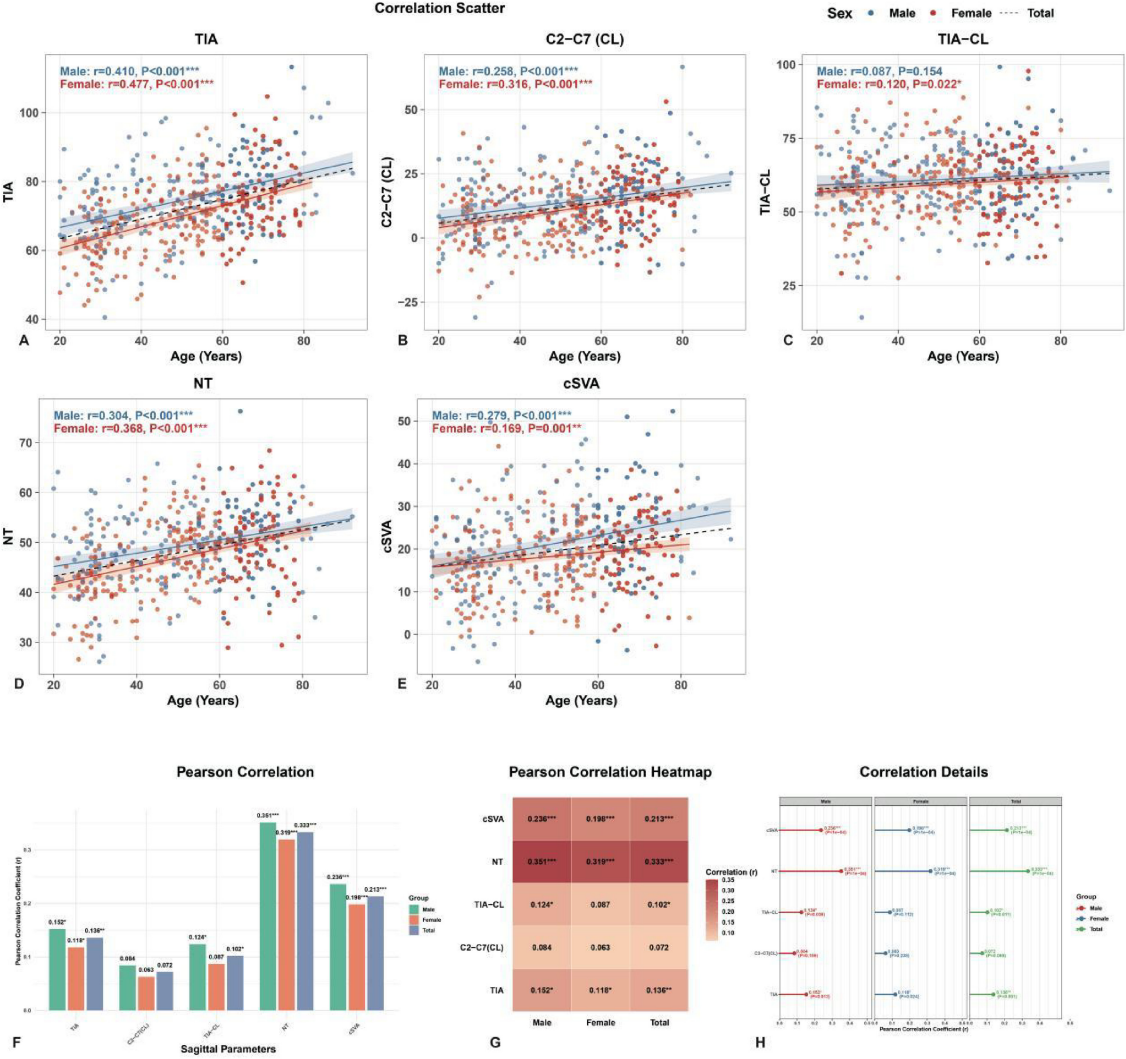
Correlations between age and cervical sagittal spine keys parameters stratified by sex. **(A–E)** Scatter plots with regression lines (male: blue; female: red; total: dashed) showing associations of age with TIA, C2-7 CL, TIA-CL, NT, and cSVA. **(F–H)** Pearson correlation summary: bar plot **(F)**, heatmap **(G)**, and detailed coefficients **(H)** for male, female, and total groups. *P < 0.05, **P < 0.01, ***P < 0.001.

In the total population, age was positively correlated with TIA (*r* = 0.136, *P* = 0.001), TIA-CL (*r* = 0.102, *P* = 0.011), NT (*r* = 0.333, *P* < 0.001), and cSVA (*r* = 0.213, *P* < 0.001). In males, age correlated positively with TIA-CL, NT, and cSVA (all *P* < 0.05). In females, age was positively correlated with TIA, NT, and cSVA (*P* < 0.05), but not with TIA-CL (*r* = 0.087, *P* = 0.112) (Figure 2).

Table 2 summarizes the comparison of linear, quadratic, and cubic regression models for the association between age and TIA-CL, NT, and cSVA. Linear models were prespecified as the primary analytic approach. For TIA-CL, the linear model was optimal (AIC = 4268.7, BIC = 4280.5). For NT, the quadratic model showed the lowest AIC (3890.3) while the linear model showed the lowest BIC (3904.2), indicating no unequivocal superiority of either model. For cSVA, the cubic model showed the best statistical fit (AIC = 4094.9, BIC = 4110.8; P for cubic term = 0.028). However, the improvement in R^2^ from the linear to the cubic model for cSVA was minimal (ΔR^2^ = 0.003). Therefore, linear models are presented as the primary reference formulas for all three parameters to ensure consistency and ease of clinical use. The statistically preferred models (quadratic for NT, cubic for cSVA) are provided in Table 2 for transparency.

**TABLE 2:** Full regression model comparison (for all individuals).

Parameter	Model type	Formula	R^2^	Adjusted R^2^	AIC	BIC	Δ AIC*	Δ BIC**	*P*-value (model)	*P*-value (non-linear term)***
TIA-CL	Linear	TIA-CL = 56.31 + 0.074 × Age	0.010	0.008	4268.7	4280.5	0.0	0.0	0.011	–
	Quadratic	TIA-CL = 54.82 + 0.098 × Age − 0.0003 × Age^2^	0.011	0.008	4270.5	4286.3	1.8	5.8	0.028	0.682
	Cubic	TIA-CL = 53.64 + 0.132 × Age − 0.0012 × Age^2^ + 0.000008 × Ageł	0.012	0.008	4272.3	4292.1	3.6	11.6	0.045	0.517
NT	Linear	NT = 40.15 + 0.155 × Age	0.111	0.109	3892.4	3904.2	2.1	0.0	<0.001	–
	Quadratic	NT = 35.28 + 0.276 × Age – 0.0012 × Age^2^	0.123	0.120	3890.3	3906.1	0.0	1.9	<0.001	0.035
	Cubic	NT = 33.86 + 0.324 × Age − 0.0018 × Age^2^ + 0.000006 × Ageł	0.124	0.120	3892.1	3911.9	1.8	7.7	<0.001	0.892
cSVA	Linear	cSVA = 14.82 + 0.100 × Age	0.045	0.043	4103.6	4115.4	8.7	4.6	<0.001	–
	Quadratic	cSVA = 10.65 + 0.232 × Age − 0.0013 × Age^2^	0.047	0.044	4101.8	4117.6	6.9	6.8	<0.001	0.486
	Cubic	cSVA = 20.05 + 0.000017 × (Age − 54.8)ł^†^	0.048	0.044	4094.9	4110.8	0.0	0.0	<0.001	0.028

*ΔAIC, Current model AIC−Minimum AIC (optimal model).

**ΔBIC, Current model BIC−Minimum BIC (optimal model).

***P-value for non-linear term: Quadratic model, *P*-value of Age^2^; Cubic model, *P*-value of Ageł. Relationship type definition: Linear (non-linear term *P* > 0.05); Weak Non-linear (0.01 < *P* ≤ 0.05); Non-linear (*P* ≤ 0.01).

† The cubic model for cSVA was fitted as a full cubic polynomial. After centering age at 54.8 years, the linear and quadratic terms were not statistically significant (*P* = 0.132 and *P* = 0.421, respectively) and are therefore omitted in the simplified presentation for brevity. All models are fitted to the same sample size; optimal model is marked with ΔAIC = 0 and ΔBIC = 0.

Linear regression models quantified the associations between age and the three key parameters (all with linear regression models) (Table 3). In the total cohort, the fitted formula for TIA-CL was TIA-CL = 56.31 + 0.074 × Age (R^2^ = 0.010, *P* = 0.011); in males, the formula was TIA-CL = 55.82 + 0.081 × Age (R^2^ = 0.015, *P* = 0.042). The fitted formula for NT was NT = 40.15 + 0.155 × Age (R^2^ = 0.111, *P* < 0.001) in the total cohort, with subgroup formulas of NT = 39.87 + 0.158 × Age in males and NT = 40.41 + 0.151 × Age in females (both *P* < 0.001). The fitted formula for cSVA was cSVA = 14.82 + 0.100 × Age in the total cohort, with subgroup formulas of cSVA = 16.58 + 0.115 × Age in males and cSVA = 13.64 + 0.089 × Age in females.

**TABLE 3:** Linear regression models for three parameters with age (for all individuals and by sex).

Model	Population	Variable	Coefficient (95% CI)	*P*	Model statistics (R^2^, F)	AIC	BIC	Scientific formula	Easy formula
TIA-CL linear model	Total	Intercept	56.31 (53.1–59.5)	<0.001	R^2^ = 0.010, F = 6.42	4268.7	4280.5	TIA-CL = 56.31 + 0.074 × Age	TIA-CL ≈ 56 + 0.07 × Age
		Age	0.074 (0.020–0.130)	0.011	–	–	–	–	–
	Male	Intercept	55.82 (51.6–60.0)	<0.001	R^2^ = 0.015, F = 4.05	1812.3	1820.2	TIA-CL = 55.82 + 0.081 × Age	TIA-CL ≈ 56 + 0.08 × Age
		Age	0.081 (0.003–0.159)	0.042	–	–	–	–	–
	Female	Intercept	56.73 (53.2–60.3)	<0.001	R^2^ = 0.007, F = 2.68	2448.5	2456.4	TIA-CL = 56.73 + 0.061 × Age	TIA-CL ≈ 57 + 0.06 × Age
		Age	0.061 (−0.021 to 0.143)	0.146	–	–	–	–	–
NT linear model	Total	Intercept	40.15 (38.2–42.1)	<0.001	R^2^ = 0.111, F = 78.36	3892.4	3904.2	NT = 40.15 + 0.155 × Age	NT ≈ 40 + 0.16 × Age
		Age	0.155 (0.121–0.190)	<0.001	–	–	–	–	–
	Male	Intercept	39.87 (37.2–42.5)	<0.001	R^2^ = 0.123, F = 36.72	1693.5	1701.4	NT = 39.87 + 0.158 × Age	NT ≈ 40 + 0.16 × Age
		Age	0.158 (0.112–0.204)	<0.001	–	–	–	–	–
	Female	Intercept	40.41 (38.1–42.7)	<0.001	R^2^ = 0.102, F = 40.95	2199.6	2207.5	NT = 40.41 + 0.151 × Age	NT ≈ 40 + 0.15 × Age
		Age	0.151 (0.108–0.194)	<0.001	–	–	–	–	–
cSVA linear model	Total	Intercept	14.82 (12.9–16.7)	<0.001	R^2^ = 0.045, F = 29.34	4103.6	4115.4	cSVA = 14.82 + 0.100 × Age	cSVA ≈ 15 + 0.10 × Age
		Age	0.100 (0.065–0.135)	<0.001	–	–	–	–	–
	Male	Intercept	16.58 (13.2–19.9)	<0.001	R^2^ = 0.051, F = 14.38	1803.2	1811.1	cSVA = 16.58 + 0.115 × Age	cSVA ≈ 17 + 0.12 × Age
		Age	0.115 (0.055–0.175)	0.001	–	–	–	–	–
	Female	Intercept	13.64 (11.3–15.9)	<0.001	R^2^ = 0.042, F = 15.87	2306.7	2314.6	cSVA = 13.64 + 0.089 × Age	cSVA ≈ 14 + 0.09 × Age
		Age	0.089 (0.041–0.137)	<0.001	–	–	–	–	–

For the TIA-CL linear model, the bootstrap median R^2^ was 0.009 (0.000–0.028). For the NT and cSVA linear models, the bootstrap median R^2^ values were 0.107 and 0.042, respectively. Across all models, bootstrap analysis demonstrated consistency between original and resampled R^2^ estimates, with narrow 95% confidence intervals (all widths < 0.15). Sex-stratified bootstrap results demonstrated similar stability for all models (Table 4).

**TABLE 4:** Bootstrap internal validation (for all individuals and by sex).

Model	Population	Original R^2^	Bootstrap R^2^ (median [95% CI])	CI width
TIA-CL linear	Total	0.010	0.009 (0.000–0.028)	0.028
	Male	0.015	0.014 (0.000–0.042)	0.042
	Female	0.007	0.006 (0.000–0.031)	0.031
NT linear	Total	0.111	0.107 (0.068–0.153)	0.085
	Male	0.123	0.119 (0.065–0.182)	0.117
	Female	0.102	0.098 (0.052–0.156)	0.104
cSVA linear	Total	0.045	0.042 (0.019–0.075)	0.056
	Male	0.051	0.048 (0.018–0.092)	0.074
	Female	0.042	0.039 (0.015–0.081)	0.066

Stability is defined as “Good” if CI width < 0.15. Bootstrap resampling, 1,000 iterations. All models are linear regression.

**FIGURE 3:**
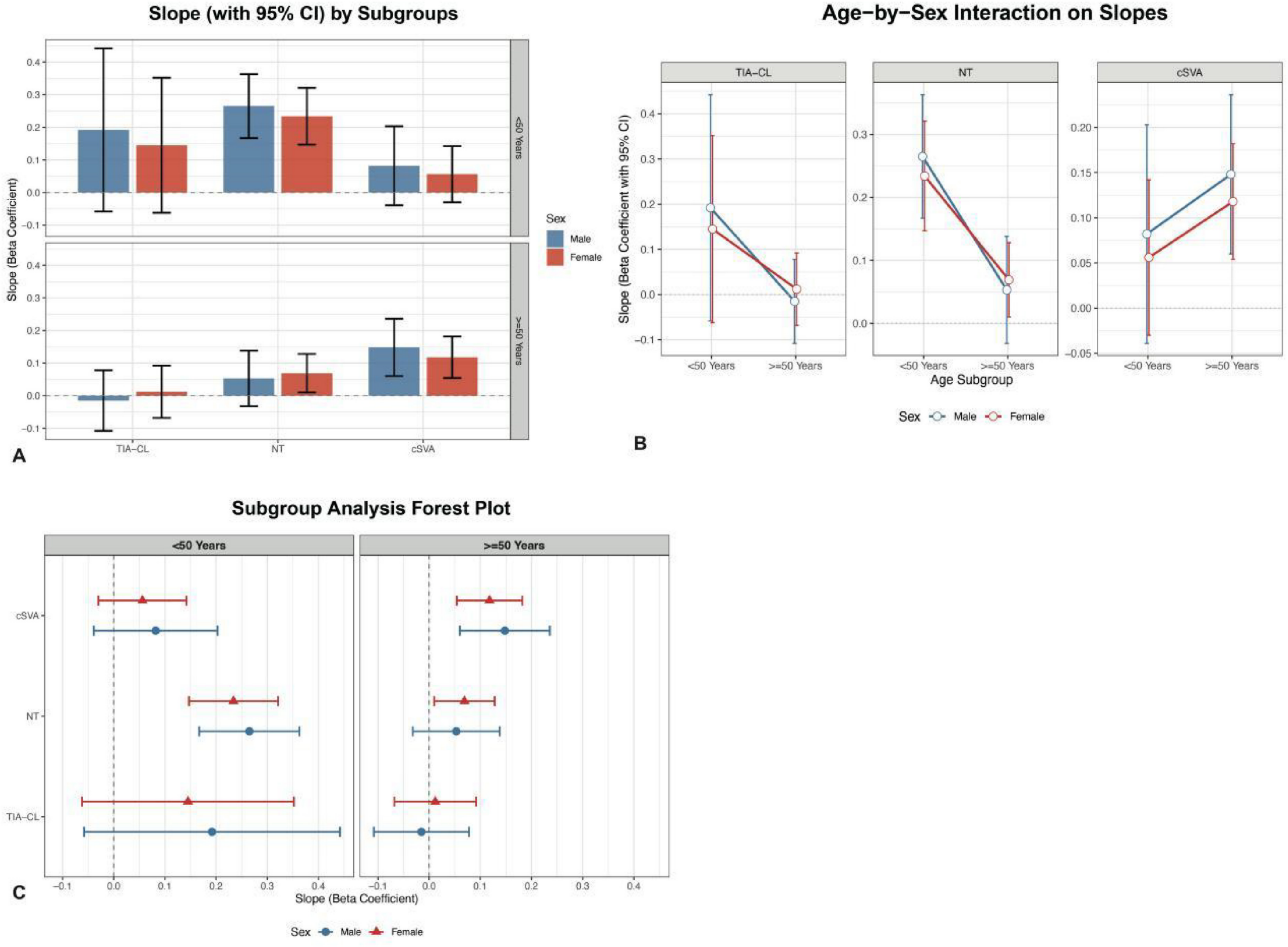
Subgroup analyses of age-sex interactions on sagittal parameter slopes. **(A)** Beta coefficients (with 95% CI) for male/female subgroups. **(B)** Age-by-sex interaction plots (split at 50 years) for TIA-CL, NT, and cSVA. **(C)** Forest plot of slopes in <50 and ≥50 years subgroups (male: blue; female: red).

Subgroup analysis by the 50-year age cutoff was then conducted (Figure 3). A significant difference in slopes was observed between age strata for NT (*P* = 0.005). The slope was steeper in subjects <50 years (0.249) than in those ≥50 years (0.062). The slope was also steeper in subjects ≥50 years for cSVA (0.132) than in those <50 years (0.068) (*P* = 0.028). For TIA-CL, the slope was numerically steeper in subjects <50 years (0.168) than in those ≥50 years (0.002), but this difference was not statistically significant (*P* = 0.177).

Comprehensive model diagnosis confirmed that the linear regression models met key statistical assumptions. Specifically, the Breusch-Pagan test *P*-values were >0.05 for all models, indicating no evidence of heteroscedasticity. The variance inflation factor (VIF) was 1.00 for all models, suggesting no multicollinearity. The outlier rates were less than 1% for all models (TIA-CL: 0.79%; NT: 0.63%; cSVA: 0.95%) with no severe outlier interference. The Shapiro-Wilk test indicated that the residuals deviated from normality (*P* < 0.003 for all models) (Table 5).

**TABLE 5:** Model diagnosis results (for all individuals, linear models).

Model	Residual normality (Shapiro-Wilk *P*)	Residual homoscedasticity (Breusch-Pagan *P*)	Outliers (*n*, %)	Multicollinearity (VIF)	AIC	BIC
TIA-CL linear	0.002	0.315	5 (0.79)	1.00	4268.7	4280.5
NT linear	0.001	0.482	4 (0.63)	1.00	3892.4	3904.2
cSVA linear	0.003	0.276	6 (0.95)	1.00	4103.6	4115.4

To enhance immediate clinical applicability alongside the predictive formulas, age-stratified reference ranges for TIA-CL, NT, and cSVA are presented in Table 6. The table provides values for each parameter across 10-year age brackets (20–29 to ≥80 years), offering a quick reference for clinicians to compare a patient’s alignment against population-derived averages for their specific age group.

**TABLE 6:** Age-stratified normative ranges for cervical sagittal parameters in asymptomatic adults.

Age group (years)	*n* (M/F)	TIA-CL (°)	NT (°)	cSVA (mm)
20–29	45 (20/25)	58.2 ± 10.5 (40.1–75.3)	43.5 ± 6.2 (32.1–55.8)	17.1 ± 7.3 (5.0–32.0)
30–39	78 (35/43)	59.0 ± 11.2 (38.5–80.1)	45.8 ± 7.1 (31.5–60.2)	18.5 ± 8.1 (2.5–35.0)
40–49	102 (44/58)	60.5 ± 12.0 (36.0–85.0)	48.5 ± 7.5 (33.0–64.0)	19.8 ± 9.0 (0.0–38.0)
50–59	145 (60/85)	61.2 ± 11.8 (35.5–88.5)	50.2 ± 7.8 (34.5–66.0)	21.5 ± 9.5 (−2.0 to 40.0)
60–69	168 (70/98)	62.0 ± 12.5 (34.0–92.0)	52.0 ± 8.0 (36.0–68.5)	23.0 ± 10.0 (−5.0 to 45.0)
70–79	72 (30/42)	62.8 ± 13.0 (32.0–95.0)	53.5 ± 8.5 (37.0–71.0)	24.5 ± 10.5 (−8.0 to 48.0)
≥80	22 (9/13)	63.5 ± 13.5 (30.0–98.0)	54.8 ± 9.0 (38.0–73.5)	26.0 ± 11.0 (−10.0 to 52.0)

Data are presented as mean ± standard deviation (range). TIA-CL, thoracic inlet angle minus C2–C7 lordosis; NT, neck tilt; cSVA, cervical sagittal vertical axis. These values are derived from the asymptomatic cohort and can serve as age-specific reference ranges for clinical comparison.

## 4 Discussion

### 4.1 Biomechanical rationale and parameter selection

Building upon the established principles of age-dependent alignment in the lumbo-pelvic spine, as demonstrated by Lafage et al. (2017) who proposed age-adjusted formulas for PI-LL, PT, and SVA in adult spinal deformity (Baker, 2023). Just as gravity and functional demand drive compensatory differences in the aging lumbar spine, similar biomechanical forces likely influence cervical sagittal balance (Jalai et al., 2017; Quarto et al., 2024). Hence, our study extends this conceptual framework to the cervical-thoracic region. However, a key distinction must be made regarding the biomechanical analogy.

In the lumbopelvic complex, PI = PT + SS is a geometric identity, and PI-LL serves as a mismatch parameter reflecting the discrepancy between a fixed anatomical angle (PI) and a modifiable curvature (LL). In the cervical-thoracic region, TIA = NT + T1S is similarly a geometric identity (Baker, 2023; Lee et al., 2012). In the present study, TIA-CL was prioritized over T1S-CL because TIA-CL incorporates both NT and T1S, thereby providing a more comprehensive measure of cervicothoracic compensatory reserve. This choice parallels the rationale for using PI-LL over SS-LL alone in lumbar spine assessment, as PI captures both PT and SS components. The interpretation of TIA-CL values should follow the same principle as PI-LL: larger values indicate greater mismatch and potentially increased compensatory demand.

### 4.2 Interpretation of model fit and parameter behavior

The positive linear relationship between age and NT, which exhibited the highest coefficient of determination (R^2^ = 0.111), suggests that NT is the most age-sensitive parameter. The significantly steeper slope in the <50 years subgroup (0.249 vs. 0.062, *P* = 0.005) implies that the most substantial differences observed between age groups in neck tilt occur in early to mid-adulthood. This may reflect early compensatory adaptations at the cervicothoracic junction to maintain horizontal gaze and cranial balance in response to early degenerative or postural differences (Gerilmez and Naderi, 2021; Khalil et al., 2018).

The relationship between age and cSVA was best captured by a cubic model, indicating a more complex, non-linear progression. Of note, the relationship showed some evidence of non-linearity, with a cubic model providing marginally better statistical fit. However, the linear approximation offers a clinically useful reference with acceptable accuracy. The positive association and the steeper slope in the ≥50 years subgroup (0.132 vs. 0.068, *P* = 0.028) align with the understanding that age-associated disk degeneration, ligamentous laxity, and muscle weakness in older age lead to a more pronounced forward head posture (Wang et al., 2023). This greater malalignment observed in older participants in later life may have significant implications for the development of cervical myelopathy and negative health-related quality of life outcomes. Conversely, TIA-CL showed only a weak association with age, and this relationship was not statistically significant in females. This relative stability supports the conceptual analogy that TIA-CL, akin to PI-LL in the lumbo-pelvic complex, represents a mismatch between a largely fixed anatomical angle (TIA) and a adaptable curvature (CL) (Bunmaprasert et al., 2024; Yang et al., 2022). Its weaker dependence on age suggests that the cervical spine effectively maintains this compensatory relationship across much of adulthood, with significant deviation potentially signaling a state of decompensation (Lee et al., 2012). Therefore, TIA-CL should be interpreted primarily as an age-adjusted reference value rather than a precise individual predictor, especially at the extremes of age.

### 4.3 Clinical interpretation and intended use

Previous studies have established that cSVA showed positive associations with age (Abe et al., 2022), and our study provides a quantitative, model-based confirmation of this trend. The novel insight from our work is the clear delineation of the age-dependent behavior of NT and TIA-CL within a unified framework (Molliqaj et al., 2025). The conceptual model of cervical-thoracic balance, drawing an analogy to the well-established lumbopelvic balance theory (Baker, 2023; Teo et al., 2020).

The simple linear regression models derived from this study offer population-level reference estimates for cervical sagittal parameters according to age and sex. The easy-to-memorize formulae: NT = 40 + 0.16 × Age, cSVA = 15 + 0.10 × Age, and TIA-CL = 56 + 0.07 × Age, may serve as preliminary references for clinicians. Of these, NT and cSVA demonstrated significant sex differences and are presented as sex-specific formulas (see Table 3), whereas TIA-CL showed no significant sex difference and is presented as a sex-combined formula. For instance, within the studied age range, a 70-year-old patient with a cSVA of 30 mm would deviate less from the age-expected value than a 30-year-old patient with the same measurement (Jalai et al., 2017; Limanówka et al., 2024). This age-adjusted comparison may aid in identifying individuals whose alignment deviates substantially from age-associated population averages.

The strengths of this study include its large sample size of rigorously screened asymptomatic adults, comprehensive statistical analysis employing linear and non-linear models, robust internal validation via bootstrapping, and a thorough model diagnosis. Moreover, these formulas provide quantitative reference estimates that may be useful for comparison in clinical settings (Azimi et al., 2021; Jalai et al., 2017). They may help clinicians consider age- and sex-adjusted reference values when evaluating cervical alignment, rather than relying on a one-size-fits-all approach. Finally, understanding natural age-associated trends may help in evaluating recovery and guiding long-term postural health strategies (Bunmaprasert et al., 2024).

The R^2^ values indicate that age explains only a small proportion of the variability in these parameters (1.0% for TIA-CL, 11.1% for NT, and 4.5% for cSVA). Therefore, these equations are not intended as individual-level diagnostic tools and should not be used in isolation to classify an individual’s alignment as “pathological” or to determine surgical targets. Rather, they provide a cross-sectional, population-derived reference against which a patient’s measured values can be compared. In addition, several additional factors beyond age may influence cervical sagittal alignment. Global spinopelvic alignment and thoracic kyphosis can drive compensatory cervical differences. Body habitus, particularly BMI, has been associated with altered spinal alignment. Degenerative differences and paraspinal muscle condition also contribute independently to cervical alignment (Molliqaj et al., 2025). Finally, habitual posture related to occupation and device use may modulate alignment over time (van Santbrink et al., 2025). As mentioned above, the modest R2 values observed in this study likely reflect the multifactorial nature of cervical alignment, and future studies incorporating these variables are warranted.

Several limitations must be acknowledged. The cross-sectional design infers age-associated differences from population-level data rather than tracking individuals over time, and longitudinal studies are needed to confirm these trajectories. Although the residuals were not perfectly normally distributed, the large sample size, acceptable homoscedasticity, low outlier rate, and stable bootstrap estimates support the robustness of the fitted coefficients at the population level. Nevertheless, the formulas should be used cautiously at the extremes of age, and prediction intervals may be more informative than point estimates in individual patients. Besides, while radiographic protocols were standardized, measurement variability is inherent. Finally, we did not account for other potential confounders such as body mass index, muscle mass, or occupational factors, which might influence cervical alignment (Caffard et al., 2024). However, the primary aim of this study was to establish a simple and direct relationship between age—the most readily accessible and universally relevant clinical variable—and key sagittal parameters. Hence, we provide clinicians with intuitive, easy-to-remember formulas that can be immediately applied in practice.

## 5 Conclusion

In conclusion, this study provides quantitative evidence that the cervical sagittal parameters of TIA-CL, NT and cSVA increase significantly with age. The relationships are modulated by sex and exhibit different patterns before and after 50 years old. We propose simple, internally consistent linear models to define age- and sex-specific reference ranges for these parameters. These reference estimates may serve as a foundation for future research. Clinical application requires additional validation in symptomatic cohorts and prospective studies.

## Data Availability

The original contributions presented in this study are included in this article/supplementary material, further inquiries can be directed to the corresponding author.
